# Induction of Reparative Dentin Formation on Exposed Dental Pulp by Dentin Phosphophoryn/Collagen Composite

**DOI:** 10.1155/2014/745139

**Published:** 2014-04-06

**Authors:** Toshiyuki Koike, Mohammad Ali Akbor Polan, Masanobu Izumikawa, Takashi Saito

**Affiliations:** Division of Clinical Cariology and Endodontology, Department of Oral Rehabilitation, School of Dentistry, Health Sciences University of Hokkaido, 1757 Tobetsu, Hokkaido 061-0293, Japan

## Abstract

The ultimate goal of vital pulp therapy is to regenerate rapidly dentin possessing an excellent quality using a biocompatible, bioactive agent. Dentin phosphophoryn (DPP), the most abundant noncollagenous polyanionic protein in dentin, cross-linked to atelocollagen fibrils was applied to direct pulp capping in rats. After 1, 2, and 3 weeks, the teeth applied were examined on the induction of reparative dentin formation and the response of pulp tissue, compared to calcium hydroxide-based agent conventionally used. The reparative dentin formation induced by DPP/collagen composite was more rapid than by calcium hydroxide. In the morphometrical analysis, the formation rate of reparative dentin by DPP/collagen composite was approximately the same as that by calcium hydroxide at 3 weeks. Nevertheless, the compactness of reparative dentin formed by DPP/collagen composite was much superior to what resulted from calcium hydroxide. Also, DPP/collagen composite showed high covering ability of exposed pulp. Moreover, DPP/collagen composite led only to slight pulp inflammation at the beginning whereas calcium hydroxide formed necrotic layer adjacent to the material and induced severe inflammation in pulp tissue at 1 week. The present study demonstrates a potential for DPP/collagen composite as a rapid biocompatible inducer for the formation of reparative dentin of excellent quality in rats.

## 1. Introduction

Direct pulp capping using calcium hydroxide-based agent has been a standard therapy for years. However, long-term studies have shown results to be variable and unpredictable [1–3]. Thus, clinical success rate of the therapy has not satisfied dentists. The agent does not provide close adaptation to dentin, does not promote consistent odontoblast differentiation, and has been shown to be cytotoxic in cell cultures, leading to the fact that the resultant reparative dentin formation can be characterized by tunnel defects [4–6]. Tunnel defects within reparative dentin may provide a pathway for the penetration of microorganisms to develop secondary infection of pulp tissue. Furthermore, low mechanical strength and pulpal resorption are potential disadvantage of the agent [5, 7]. Therefore, a biocompatible, bioactive agent that rapidly induces reparative dentin formation possessing a good quality is required to improve the clinical success rate of vital pulp therapy.

Dentin phosphophoryn (DPP), a member of small integrin-binding ligand N-linked glycoproteins (SIBLING) family, is the most abundant of the noncollagenous polyanionic proteins in dentin. DPP is dentin sialophosphoprotein (DSPP) gene product [8, 9]. It is predominantly expressed in odontoblasts and is known to be a marker of the differentiation of pulp cells into odontoblasts [10–12]. It contains the RGD motif at position of 26 from N-terminal and the repeating sequence of (asparatic acid-phosphoserine-phosphoserine)_n_ as its characteristic domains [13]. Aspartic acid and serine account for at least 75% of all amino acid sequences, and 85%–90% of the serine residues are phosphorylated. DPP is supported by* in vitro *mineralization data showing that DPP is an important initiator and modulator for the formation and growth of hydroxyapatite crystals due to many negatively charged regions [14–19]. In particular, DPP covalently cross-linked on collagen fibrils has high potential to nucleate hydroxyapatite according to the measure of interfacial energy [17–19]. Recently, DPP has been reported to induce the differentiation of human mesenchymal stem cells into osteoblasts as its RGD motif binds with *α*v*β*3 integrin on the cell surface followed by the activation of the MAP kinase and SMAD signaling pathway [20, 21]. Moreover, we demonstrated that DPP promotes not only differentiation or calcification but also cell migration by acting on integrin on the surface of pulp cells via its RGD motif* in vitro *[22]. However, the potential of DPP for dentin regeneration in animal model has not still been clear although there is much evidence on DPP* in vitro *ability.

The aim of this study was to investigate effects of DPP cross-linked to type I atelocollagen fibrils as a scaffold placed on experimentally created pulp exposures in first molar in rats on induction of reparative dentin formation and response of pulp tissue, compared to calcium hydroxide-based agent conventionally used.

## 2. Materials and Methods

### 2.1. Preparation of DPP Cross-Linked to Type I Atelocollagen Fibrils

The DPP was prepared from molar teeth extracted from 8-9-month-old porcine jaws following the method described by Butler [23]. The preparation was carried out at 4°C in solutions to which protease inhibitors (100 mM 6-aminohexanoic acid, 5 mM benzamidine-HCl, and 1 mM phenylmethylsulfonyl fluoride) were added. The DPP was extracted with calcium precipitation method and purified through the anion exchange chromatography step.

Type I atelocollagen fibrils, from which telopeptides known to be antigen had been removed, were used as a carrier of DPP in this study. The DPP was cross-linked to porcine-derived type I atelocollagen fibrils (collagen sponge, Nitta gelatin, Osaka, Japan) with divinylsulfone (Sigma Chemical, St. Louis, MO, USA) [24]. To remove unreacted divinylsulfone and DPP that was not covalently bound, the substrates were washed with 0.5 M NaCl, 0.05 M Tris-HCl, and pH 7.4, ten times and then washed with distilled water. The composite was analyzed for phosphate analysis [25] after alkaline hydrolysis to determine the amount of DPP cross-linked to type I atelocollagen fibrils.

### 2.2. Surgical Procedure

All animal procedures were in accordance with the guidelines of the Animal Care Committee of the Health Sciences University of Hokkaido. Eight-week-old male Wistar rats (Hokudo, Sapporo, Japan) were used in this study. Animals were anaesthetized with an intraperitoneal injection of pentobarbital (40 mg/kg). The vital pulp tissue was exposed by drilling the mesial buccal cusp of the maxilla first molar using a sterile, round steel bur (number 014, Dentsply, York, PA, USA). The exposed pulp tissue was treated with 10% sodium hypochlorite and 3% hydrogen peroxide. Then, bleeding was controlled with sterile cotton pellets. Subsequently, the exposed pulp tissue was covered with 0.5 *μ*g of DPP covalently cross-linked to 29.5 *μ*g of type I atelocollagen fibrils (DPP-Col), type I atelocollagen alone (Col), and multi-Cal (Ca) (Pulpdent, Watertown, MA, USA), one of calcium hydroxide-based agents which have been conventionally used for direct pulp capping. Twenty-one teeth were used in each group (DPP-Col, Col, and Ca) and were further divided into three groups on the basis of experimental period (1, 2, and 3 weeks). Following the pulp capping, each cavity was immediately sealed with glass-ionomer cement (Hy-bond Glasionomer CX, Shofu, Koto, Japan) and was filled with single step bonding system (Clearfil S-3 Bond, Kuraray, Tokyo, Japan) and composite resin (Unifil flow, GC, Tokyo, Japan).

### 2.3. Histological Examination

At 1, 2, and 3 weeks after surgery, the animals were killed by overinhalation of diethyl ether. Following death, all experimental teeth and the adjacent alveolar bone were removed and fixed with 10% neutral buffered formalin for 24 hours. Then, specimens were demineralized in 0.1 M EDTA, pH 7.4, at 4°C, embedded in paraffin, sectioned at 4 *μ*m, and subsequently stained with hematoxylin and eosin. The sections were then analyzed in a light microscope (Eclipse E400, Nikon, Tokyo, Japan).

### 2.4. Morphometrical Analysis of Reparative Dentin Formed and Evaluation of Pulp Tissue

First, the formation rate of reparative dentin, that is, relative area of reparative dentin formed adjacent to the cavity to the area of pulp chamber of crown in each experimental tooth, was assessed in the longitudinal section covering the central part of the experimental wound. The areas in the sections were measured by means of Image J (Wayne Rasband, MD, USA) in three sections in separate seven teeth. Next, the compactness of reparative dentin formed in the sections was measured based on the area of defect in reparative dentin and the total area of reparative dentin. The formation rate and compactness of reparative dentin were calculated for formula as follows [26]:

 Formation rate (%) = total area of tertiary dentin/coronal pulp chamber area × 100
 Compactness (%) = 1 − (defects area + cells area)/total area of tertiary dentin × 100.

Then, the covering degrees of exposed pulp tissue with reparative dentin formed were assessed in four scores; 4: completely covered, 3: almost covered (50% and over covering at the pulp exposure surface), 2: partially covered (49% and less covering at the pulp exposure surface), and 1: reparative dentin formation not observed. Moreover, the degrees of pulp inflammation induced by the materials were evaluated in four scores; 4: minimal inflammation (none or few scattered inflammatory cells present in the pulp at the exposure site, or same as normal dental pulp), 3: mild inflammation (some vasodilation of blood vessels, indicating mild hyperemia on the surface of the exposure site), 2: moderate inflammation (presence of weak vasodilation of blood vessels without infiltration of blood cells into the dental pulp, some inflammatory cells, such as polymorphonuclear leukocytes and neutrophils, observed), 1: severe inflammation (presence of strong vasodilation of blood vessels appearing as an abscess and significant inflammatory infiltration by polymorphonuclear leukocytes and neutrophils seen throughout the crown) [26].

### 2.5. Statistical Analysis

The results for the formation rate, the compactness of reparative dentin formed, and the degrees of pulp inflammation induced by the materials are expressed as means ± SD. They were analyzed by one-way analysis of variance with Tukey's multiple comparison test. Differences at *P* < 0.05 were considered statistically significant.

## 3. Results

**Figure 1:**
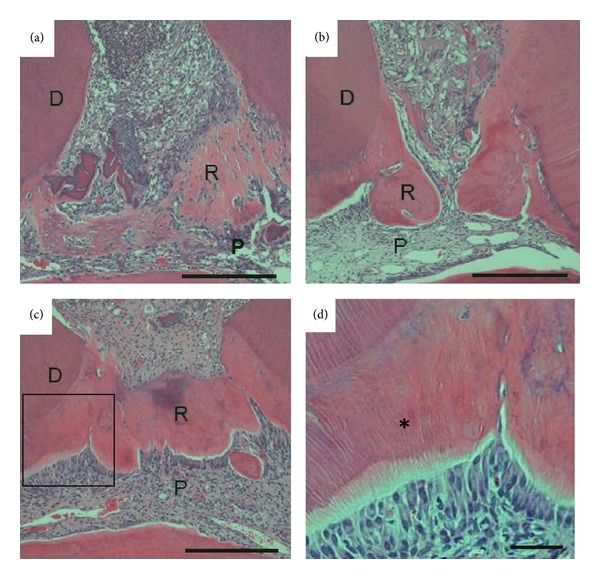
Micrographs showing upper first molars 1(a), 2(b), and 3 weeks ((c) and (d)) after treatment with DPP-Col. The compact reparative dentin was formed adjacent to the primary dentin at 2 weeks (b). The exposed pulp tissue was covered completely with reparative dentin at 3 weeks (c). Reparative dentin has dentinal tubules (asterisk) and regular arrangement of odontoblasts at 3 weeks ((d) higher magnification of the highlighted region in (c)). D: dentin, P: pulp tissue, and R: reparative dentin. Scale bar ((a), (b), and (c)) = 500 *μ*m. Scale bar (d) = 100 *μ*m.

**Figure 2:**
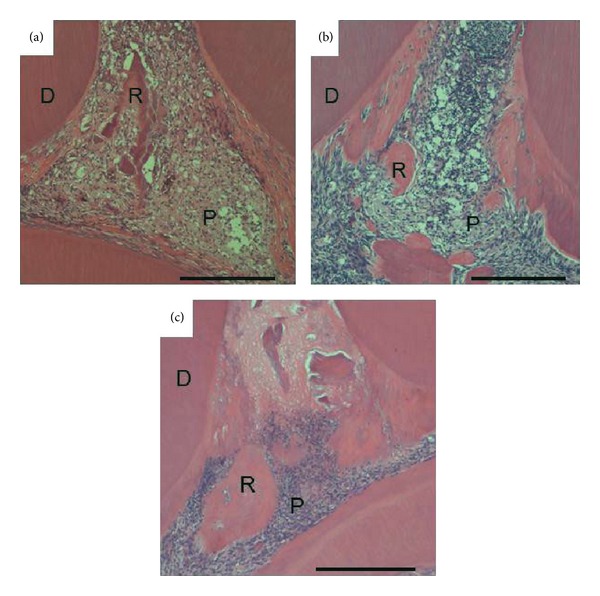
Micrographs showing upper first molars 1(a), 2(b), and 3 weeks (c) after treatment with Col. Reparative dentin is observed at 2 weeks but does not cover exposed pulp tissue even at 3 weeks. D: dentin, P: pulp tissue, and R: reparative dentin. Scale bar = 500 *μ*m.

**Figure 3:**
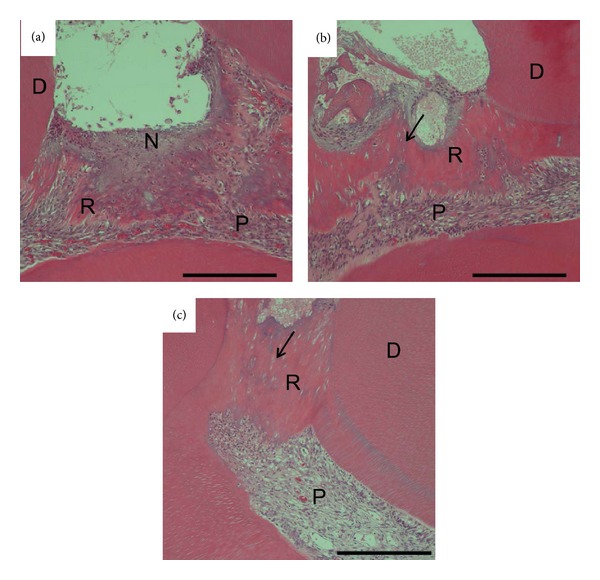
Micrographs showing upper first molars 1(a), 2(b), and 3 weeks (c) after treatment with Ca. Reparative dentin having gaps and tunnel defects was observed at 2 weeks (arrow). This dentin still contained gaps and tunnel defects at 3 weeks. A necrotic superficial layer (N) formed beneath the material. D: dentin, P: pulp tissue, and R: reparative dentin. Scale bar = 500 *μ*m.

**Figure 4:**
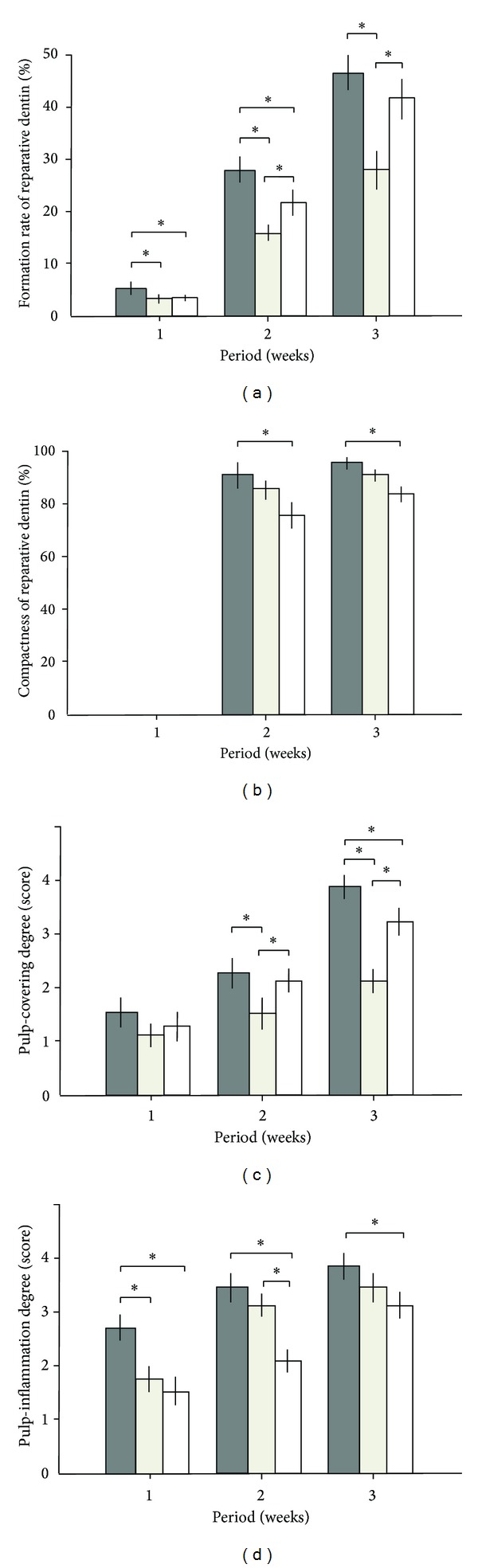
Evaluation of the reparative dentin and dental pulp tissue. (a) Formation rate of the reparative dentin (%), (b) compactness of the reparative dentin (%), (c) degree of coverage by the reparative dentin (score), and (d) inflammation degree of the dental pulp tissue (score). Gray columns: DPP-Col, beige columns: Col, and white columns: Ca. Twenty-one teeth were used in each group (DPP-Col, Col, and Ca) and were further divided into three groups on the basis of experimental period (1, 2, and 3 weeks). *P < 0.05.

The formation rate of reparative dentin by every group increased with time (Figures 1–3). In DPP-Col group, the formation rate of reparative dentin was significantly higher, compared with that by Col group throughout the experimental period (Figure 4(a)). It was also significantly higher than that by Ca group at 1 and 2 weeks. However, no significant difference was noted at 3 weeks between groups DPP-Col and Ca. In addition, the compactness of reparative dentin formed by DPP-Col group was significantly higher, compared with that formed by Ca group at 2 and 3 weeks (Figure 4(b)). In particular, it showed approximately 97% at 3 weeks. In DPP-Col group, reparative dentin possessing dentinal tubules was formed adjacent to the primary dentin at 3 weeks (Figures 1(c) and 1(d), asterisk). The exposed pulp tissue was covered completely with reparative dentin (Figures 1(c) and 4(c)) having regular arrangement of odontoblasts at 3 weeks (Figure 1(d)). The slight pulp inflammation was observed at 1 and 2 weeks, but no pulp inflammation was observed at 3 weeks (Figures 1 and 4(d)).

In Col group, reparative dentin formation was observed at 2 weeks, but reparative dentin did not cover exposed pulp tissue at even 3 weeks (Figures 2 and 4(c)). Moreover, the mild pulp inflammation was observed only at the early stage after operation (Figure 4(d)).

In Ca group, reparative dentin having considerable amount of gaps and tunnel defects was observed at 2 weeks (Figure 3(b), arrow). At 3 weeks, the reparative dentin covered exposed pulp tissue but still contained gaps and tunnel defects (Figure 4(c)). The arrangement of odontoblasts underneath reparative dentin formed by Ca group was not clear. The formation of necrotic layer adjacent to the material and severe inflammation in pulp tissue were observed at 1 week (Figure 3(a)). The mild pulp inflammation still remained at 3 weeks (Figures 3(c) and 4(d)).

## 4. Discussion

It has been reported in clinical studies that the success rate of direct pulp capping with calcium hydroxide that has been used conventionally is approximately 60% even though it has been considered the standard therapy [27]. The clinical failures of direct pulp capping have led to the search for new therapeutic agents. The potential of bioactive agents such as dentin extracellular matrix molecules, for example, BMP-2, BMP-4, and BMP-7 (osteogenic protein-1: OP-1), dentin matrix protein-1 (DMP-1), matrix extracellular phosphoglycoprotein (MEPE), bone sialoprotein (BSP), and so forth, enamel matrix derivative, or stem cells is currently under study [11, 28–34]. Recently, mineral trioxide aggregate (MTA) was found to be an excellent pulp-capping agent because of its high biocompatibility [35]. However, dentin bridge formation by MTA was shown to be slower compared to calcium hydroxide [36]. In the clinical case, exposed pulp should be closed by the complete dentin bridge in a short period. The dentin extracellular matrix molecules are promising materials because of being involved in dentinogenesis during the development of teeth [37]. Therefore, they can bring out the potential of stem cells or progenitor cells located within the pulp, to the maximum, to proliferate, differentiate into odontoblast-like cells, and consequently produce the extracellular matrix, which will ultimately undergo mineralization.

In the present study, we confirmed DPP's high ability for dentin regeneration in animal model. After direct pulp capping with DPP cross-linked to type I collagen, biocompatible scaffold, the formation of reparative dentin was more rapid at the beginning than with calcium hydroxide. The rapid induction of reparative dentin reduces the chance of secondary infection of the pulp. No significant difference was found in the formation rate of reparative dentin between DPP cross-linked to collagen and calcium hydroxide at 3 weeks after operation. However, the compactness of reparative dentin formed by DPP cross-linked to collagen was significantly higher compared with that formed by calcium hydroxide at 2 and 3 weeks. In particular, the porous reparative dentin having tunnel defect was formed by calcium hydroxide. It has been reported that 89% of dentin bridges formed by calcium hydroxide contained tunnel defects [5]. The tunnel defects fail to provide a hermetic seal to the underlying pulp against recurring infection due to microleakage. This reparative dentin has a bone-like structure that does not possess odontoblast layer underneath it and consequently does not contain dentinal tubules. In contrast, DPP immobilized to type I collagen induced the compact reparative dentin having regular arrangement of odontoblasts and dentinal tubules. This accounts for the quality difference of reparative dentin formed between DPP immobilized to type I collagen and calcium hydroxide. Thus, it was suggested that DPP induced differentiation from undifferentiated pulp cells into odontoblasts on type I collagen fibrils, suitable scaffolds for the cell differentiation, after the activation of the MAP kinase and SMAD signaling pathway [20, 21].

DPP immobilized to type I collagen caused only slight pulp inflammation at the early stage after operation due to the biocompatibility of collagen. The pulp exposure and implantation onto the pulp of biomaterials induce inflammation. Many studies report that the healing sequence includes an initial inflammation process [38–40]. In inflammatory phenomena, pulp cells express class II antigens [41]. They are implicated in the immune reaction. Immune cells such as dendritic cells and macrophages are crucial in the control of cell proliferation and apoptosis [42]. They contribute in the exposed pulp to resolve inflammatory process [43]. Then, the reparative process follows after inflammation. Therefore, inflammatory reaction may be another prerequisite for the repair process of dentin/pulp complex. On the other hand, calcium hydroxide formed necrosis layer underneath the agent due to its high alkalinity and also caused pulp inflammation. It has been reported that 41% of them were associated with recurring pulp inflammation or necrosis [5]. Thus, calcium hydroxide cannot be considered as a biocompatible agent although it is often used as a convenient low-cost material for vital pulp therapy.

In the present study, divinylsulfone was used for covalent cross-linking of DPP to type I collagen fibrils. It has been reported as a cross-linker of biomaterials* in vitro *and* in vivo *[44, 45]. It is contained in the osteoarthritis knee pain relief treatment agent for clinical use [46]. Thus, DPP/collagen composite can be considered as a safe material. For clinical application of DPP/collagen, however, unreacted divinylsulfone should be removed from the composite completely after cross-linking procedure, due to its toxicity [47].

This is the first time that DPP/collagen composite showing stimulation of reparative dentin formation was reported. Our observation might help develop a reliable and safe vital pulp therapy. However, it is necessary to develop artificial materials such as recombinant protein and synthetic peptide of DPP for clinical trial for avoiding possible immune reaction and contamination of unidentified factors. Also, more investigations in long-term experiments will reveal more characteristics of DPP/collagen composite for dentin regeneration, and the further refinement of the collagen scaffold is necessary in order to utilize maximally the potential of DPP to stimulate the formation of reparative dentin as a hard tissue barrier. Moreover, it is crucial to understand the mechanisms underlining the process of pulp repair as well as dentin regeneration.

## 5. Conclusion

The present study demonstrates a potential for DPP/collagen composite as a rapid biocompatible inducer for the formation of reparative dentin of excellent quality in rats. Therefore, it is a promising biocompatible pulp capping material for the future.
